# Contributions of Pharmacovigilance to the Understanding of Risks Associated with Ibuprofen: Descriptive and Disproportionality Analysis Using FAERS Data

**DOI:** 10.3390/ph19020319

**Published:** 2026-02-14

**Authors:** Cristina Anamaria Buciuman, Carmen Maximiliana Dobrea, Anca Butuca, Adina Frum, Felicia Gabriela Gligor, Mihai Octavian Botea, Mariana Eugenia Mureșan, Octavia Gligor, Florin Maghiar, Luciana Dobjanschi, Otilia Micle, Claudiu Morgovan, Laura Grațiela Vicaș

**Affiliations:** 1 Faculty of Pharmacy, Carol Davila University of Medicine and Pharmacy, 6 Traian Vuia Str., 020956 Bucharest, Romania; buciuman.apetrii@drd.umfcd.ro; 2 Preclinical Department, Faculty of Medicine, “Lucian Blaga” University of Sibiu, 550169 Sibiu, Romania; carmen.dobrea@ulbsibiu.ro (C.M.D.); anca.butuca@ulbsibiu.ro (A.B.); adina.frum@ulbsibiu.ro (A.F.); felicia.gligor@ulbsibiu.ro (F.G.G.); claudiu.morgovan@ulbsibiu.ro (C.M.); 3 Faculty of Medicine and Pharmacy, University of Oradea, 10, 1 December Square, 410073 Oradea, Romania; mbotea@uoradea.ro (M.O.B.); mmuresan@uoradea.ro (M.E.M.); fmaghiar@uoradea.ro (F.M.); dobjanschil@yahoo.com (L.D.); micleotilia@gmail.com (O.M.); lvicas@uoradea.ro (L.G.V.)

**Keywords:** ibuprofen, FAERS, pharmacovigilance, ibuprofen safety profile, descriptive analysis, disproportionality analysis

## Abstract

**Background/Objectives:** The objective of this study was to evaluate real-world evidence (Food & Drug Administration database, FAERS) on ibuprofen adverse events (AE) through descriptive and disproportionality analyses. **Methods:** Signal assessment involved analyzing the top 30 entries with the most reports. The disproportionality analysis of signals based on Evans’ criteria (number of reports > 2, chi-square > 4, and PRR > 2) was performed. A total of 70,792 reports submitted to FAERS by the end of 2024 (collected from 97 countries worldwide) indicate ibuprofen as the main suspect. **Results:** Of these, the highest percentage was attributed to females (n = 33,262, 47.0%) and adult patients (18–65 years) (n = 22,005, 31.1%). In the elderly group (12.4%) and in children and adolescents (11.2%), similar frequencies were reported. Oral administration was the most frequently mentioned route (n = 25,035, 35.4%). A total of 21,077 reports had an unfavorable outcome, of which 3018 (4.3%) reported death. **Conclusions:** The results highlight potential risks associated with ibuprofen and emphasize the importance of responsible, clinically well-founded administration. The disproportionality analysis can provide valuable information for effectively selecting drug-adverse-effect pairs that warrant further attention.

## 1. Introduction

Ibuprofen belongs to the class of nonsteroidal anti-inflammatory drugs (NSAIDs), and it is an over-the-counter (OTC) medication or, in some cases, is dispensed based on a medical prescription. In the United States (US) of America, in 2022, ibuprofen was prescribed to more than 9 million patients, a 7% increase from 2021 [1]. The administration routes used are oral, cutaneous, rectal or intravenous [2,3,4]. Its formulation implies adapting to the specific pharmaceutical formulations via techniques that facilitate the effective delivery of the active substance [5]. In the US, over 40 products containing ibuprofen, under different pharmaceutical formulations, are included in the US Food & Drug Administration (FDA) Adverse Event Reporting System (FAERS) database [6].

Recent epidemiological data suggest that the escalating use of ibuprofen is not only driven by its analgesic efficacy but also by its expanding role in managing chronic inflammatory conditions in an aging population. This increased exposure needs a rigorous re-evaluation of its safety through large-scale real-world data, as clinical trials often lack the power to detect rare or long-term complications [7,8,9].

Ibuprofen’s main mechanism of action involves the nonselective inhibition of the group of cyclooxygenase enzymes (COX 1–3) [8]. Thus, the conversion of the arachidonic acid into prostaglandins—key mediators of pain and inflammation—is blocked [10]. Generally well tolerated, ibuprofen has been associated over the years with ADRs of gastro-intestinal [11], cardiovascular [12,13,14], cutaneous [12], renal [15], and neuropsychiatric [16,17] nature.

Furthermore, recent studies have highlighted the complexity of NSAID-induced adverse events, emphasizing that the risk profile is highly individualized. Emerging evidence points towards a significant interplay between genetic predispositions and the frequency of severe reactions, such as drug-induced liver injury or complex hypersensitivity syndromes, which remain under-reported in traditional clinical settings [18,19,20,21].

ADRs are collected and monitored by the authorities throughout databases. The monitoring system comprises reports from the pharmaceutical industry, generated by healthcare professionals, alongside patients. The terms utilized in reporting are part of the standardized Medical Dictionary for Regulatory Activities (MedDRA) [22]. Reporting is mandatory for drug manufacturers, voluntary for healthcare professionals (doctors, pharmacists, and nurses), and voluntary for patients who notice a possible ADR. This type of database can provide insight into a drug’s real-world safety profile. It can be used to continuously monitor and evaluate AEs and discover new ADRs, especially rare ones.

The FAERS database is a cornerstone of “real-world” drug safety, being frequently used in post-marketing studies. Disproportionality analysis is a validated statistical approach recommended in pharmacovigilance studies for identifying signals by comparing the observed frequency of a drug–event combination with a background frequency [23].

This methodology is particularly effective for widely used drugs like ibuprofen, where even low-frequency events can have a substantial public health impact due to the sheer volume of users [24,25].

The objective of the present study was to evaluate real-world evidence (the FAERS database) on ibuprofen safety, with the aim of examining the need for any updates to its safety profile, as known to date. By employing both descriptive and disproportionality analyses, this research seeks to bridge the gap between clinical trial data and everyday clinical practice, ensuring that the safety information remains current and evidence-based.

## 2. Results

### 2.1. Descriptive Analysis of Reported Events

**Figure 1:**
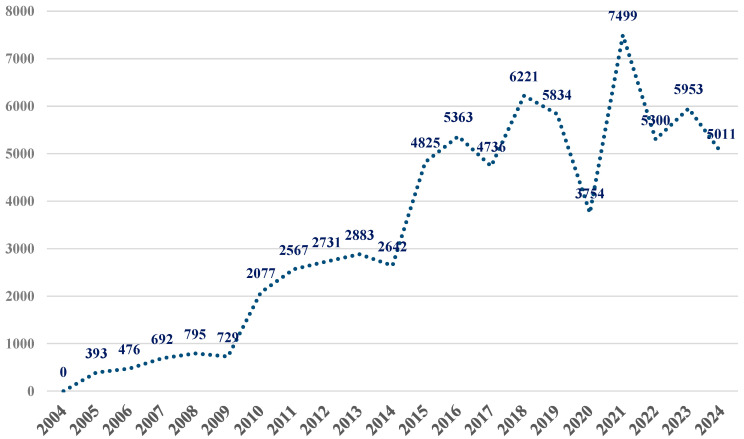
Evolution of reports for ibuprofen in the FAERS database, period of 2004–2024 (n = 70,792).

In FAERS, a total of 70,792 reports were identified in association with ibuprofen as the primary suspect drug. Figure 1 shows that the number of reports increased steadily from 2004, reaching a peak in 2021 (n = 7499), followed by a slight decline in 2022–2024.

**Figure 2:**
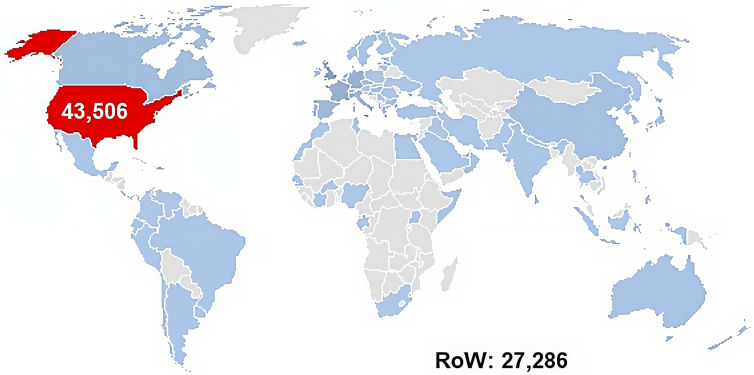
Country-level distribution of ibuprofen case reports submitted to FAERS as of 31 December 2024 (n = 70,792). RoW = Rest of the World. Red—the highest number (United States of America); Blue—the intensity of the blue shade is proportional to the report number for Rest of the World; grey—no reports registered in FAERS.

Figure 2 reveals these reports originated from 97 countries worldwide, and 1.14% of them did not have the country of provenance mentioned (n = 809). As expected, 61.5% were documented in the US, FAERS being managed by the FDA. Among the remaining reports, the largest contributions came from Western European countries: Great Britain (n = 6012, 8.5%), Germany (n = 3791, 5.4%), France (n = 3772, 5.3%), Italy (n = 2277, 3.2%), Spain (n = 2230, 3.2%), Portugal (n = 810, 1.1%), and the Netherlands (n = 500, 0.7%). Other notable contributions were from Canada (n = 1446, 2.0%), Australia (n = 563, 0.8%), and China (n = 1048, 1.5%). This geographic distribution highlights the predominance of reporting from North America and Western Europe, likely reflecting the national character of FAERS database as well as the reach of pharmacovigilance systems and reporting practices in these regions.

**Figure 3:**
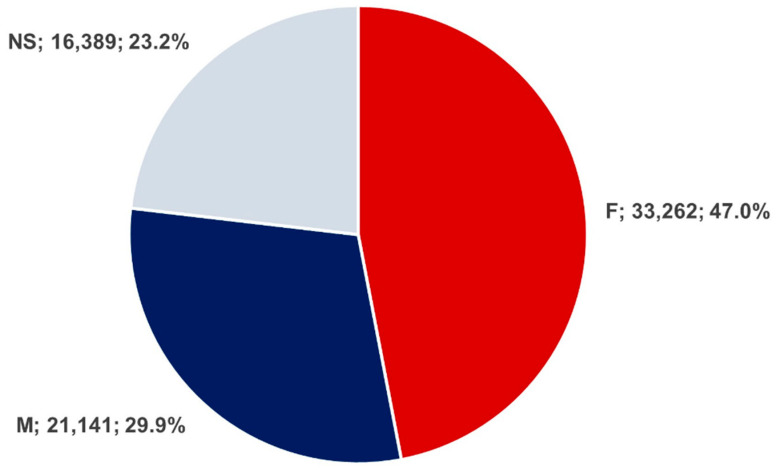
Sex distribution for reports concerning ibuprofen (n = 70,792). F—female patients, M—male patients, NS—not specified.

Among the deduplicated cases, 47.0% were female (n = 33,262) and 29.9% were male (n = 21,141), while in 23.1% of reports the patient’s sex was not specified or was reported as unknown (n = 16,389). As shown in Figure 3, this distribution reflects the known limitations of spontaneous reporting systems such as FAERS.

**Figure 4:**
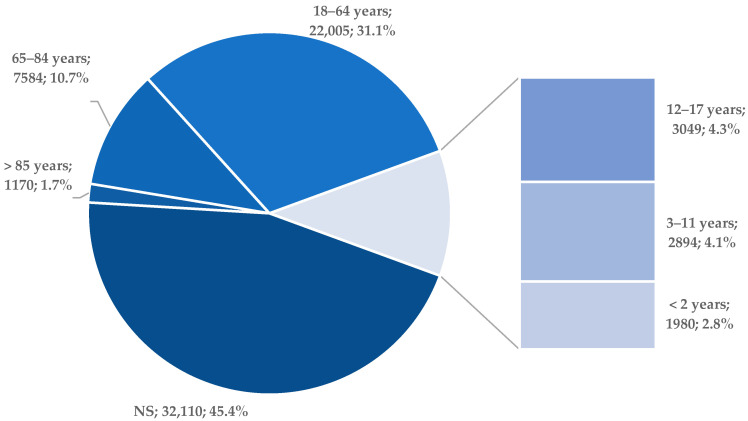
Case distribution based on the age group of patients (n = 70,792). NS—not specified.

Figure 4 shows the age distribution of ibuprofen-associated adverse event reports in FAERS. Approximately 11.0% of cases involved children and adolescents, while 12.4% were reported for elderly individuals over 65 years old. As expected, the majority of reports were for adults aged 18–64 (n = 22,005, 31.1%). However, a large proportion of cases (45.4%) lacked information on patient age. Therefore, the age-stratified analysis reflects only a subset of the dataset and may not fully represent the true age distribution of ibuprofen-associated adverse events.

Table 1 displays the administration routes mentioned in the reports recorded in FAERS for ibuprofen. A large number of reports did not contain the administration route (n = 45,020, 63.6%). The oral route was the most frequently specified (n = 25,035, 35.4%). The transplacental (n = 367, 0.5%) and transmammary (n = 13, 0.02%) routes were notable, suggesting the occurrence of adverse events in newborns. Of the parenteral routes, the intravenous route (n = 153, 0.22%) was the most reported and, consequently, was the second most reported of these routes.

**Figure 5:**
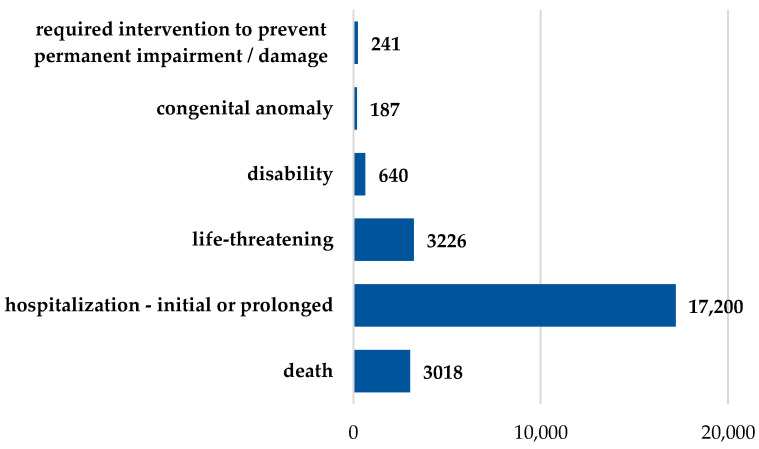
Unfavorable outcomes associated with ibuprofen administration recorded in FAERS (n = 24,512).

Adverse events of ibuprofen reported having an unfavorable outcome represented approximately 30%. Hence, at least one unfavorable outcome was reported for 21,077 cases. Death was reported for 3018 patients (4.3%). According to Figure 5, initial or prolonged hospitalization of the patient was required for 24.3% of the cases (n = 17,200). A life-threatening event was reported in 4.6% of the cases (n = 3226), and 640 cases (0.9%) led to a disability. Remarkably, a congenital anomaly was linked to ibuprofen in 187 cases (0.3%). Lastly, medical intervention in order to prevent permanent impairment or damage of the patient’s condition was necessary in 241 cases (0.3%).

### 2.2. FAERS Recorded Signal Assessment

A total of 176,356 adverse events were reported for the 4826 signals. Analyzing the top 30 signals with the most reports (representing a total of 56,546 events), most events were: product use in unapproved indication (n = 3569), drug hypersensitivity (n = 3407), drug effective for unapproved indication (n = 2459), and vomiting (n = 2447) (Table 2). A total of 8772 reports (5.00% of the dataset) indicated a lack of therapeutic effect (drug ineffective). These reports are presented here descriptively to provide context regarding ibuprofen use and patient-reported outcomes.

Based on the disproportionality analysis, only 17 of the first 30 signals, in terms of number of reported events, were possible ADRs for ibuprofen. These were categorized as:

- Gastrointestinal disorders: abdominal pain upper, abdominal discomfort, and gastrointestinal hemorrhage;
- Immune system disorders: drug hypersensitivity, urticaria, angioedema, and anaphylactic reaction;
- Renal disorders: acute kidney injury;
- Psychiatric disorders: somnolence and suicide attempt;
- Injuries, intoxications, and procedural complications: product use in unapproved indication, drug effective for unapproved indication, overdose and intentional overdose, and toxicity to various agents.

Among these 17, the 5 most frequent ADRs with clinical manifestations were: drug hypersensitivity (ROR: 4.85, 95% CI: 4.69–5.03; PRR: 4.67; Χ^2^: 9675.03), urticaria (ROR: 3.88, 95% CI: 3.70–4.06; PRR: 3.80; Χ^2^: 3722.68), acute kidney injury (ROR: 3.93, 95% CI: 3.75–4.12; PRR: 3.86; Χ^2^: 3713.88), abdominal pain upper (ROR: 2.82, 95% CI: 2.68–2.96; PRR: 2.78; Χ^2^: 1850.02), and angioedema (ROR: 11.54, 95% CI: 10.97–12.13; PRR: 11.29; Χ^2^: 14,491.17).

Nonetheless, for some reported events, the possibility of these being ADRs was unlikely (false signals), although they are mentioned in the Prescribing Information of ibuprofen-containing products: nausea, vomiting, dyspnea, rash, pruritus, dizziness, and headaches.

**Figure 6:**
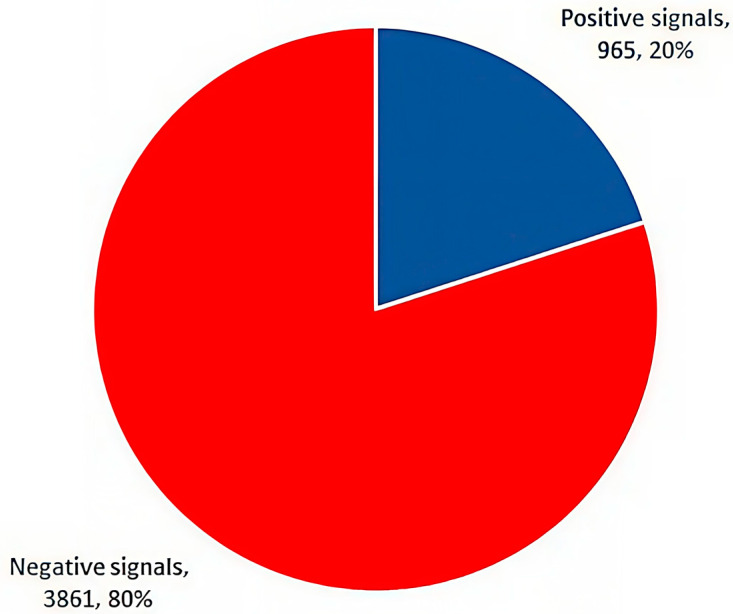
Classification of signals according to their probability of correlation with ADRs (n = 4826).

Of the total of 4826 signals recorded for ibuprofen in FAERS, 20.0% (n = 965, 77,329 reports) were positive signals, while the remaining 80.0% (n = 3861, 99,027 reports) were classified as false signals (Figure 6).

The strongest positive signals were defined as those with the highest chi-square values (Table 3). Considering this criterion, the top 30 strongest positive signals included adverse drug reactions (ADRs) from the following categories:

- Gastrointestinal: seven signals (gastric hemorrhage, oral discomfort, duodenal ulcer, upper gastrointestinal hemorrhage, melaena, hematemesis, and gastric ulcer);
- Allergic: six signals (toxic epidermal necrolysis, urticaria, anaphylactic reaction, lip oedema, drug hypersensitivity, and angioedema);
- Procedural complications: five signals (product administered to patient of inappropriate age, accidental exposure to the product by child, intentional overdose, product use in an unapproved indication, and drug effective for an unapproved indication);
- Renal: four signals (minimal change glomerulonephritis, acute kidney injury, tubulointerstitial nephritis, and renal tubular acidosis);
- Respiratory: three signals (bronchopulmonary dysplasia, aspirin-exacerbated respiratory disease, and NSAID-exacerbated respiratory disease);
- Product-related issue: two signals (failure of child-resistant product closure and poor-quality drug administered);
- Metabolic: one signal (metabolic acidosis);
- Neurological: one signal (aseptic meningitis);
- Psychiatric: one signal (suicide attempt).

## 3. Discussion

The present study showed that, in FAERS database, a total of 70,792 reports were identified in association with ibuprofen as the primary suspect drug. The evolution of reports up to the end of 2024 shows an upward trend, with a peak in 2021 (when more than 10% of total reports from the past 20 years were recorded). Similar patterns have been reported in other pharmacovigilance studies [23]. All these suggest that temporary drops in reporting may reflect changes in prescribing or in the drug’s use. Therefore, FAERS data must be interpreted as reflecting reporting trends rather than actual incidence rates.

Ibuprofen is among the most widely used NSAID drugs. The high number of adverse events reported in FAERS could be attributed to its over-the-counter availability. Also, the widespread access to ibuprofen in diverse settings, frequently without medical supervision, can elevate the risk and reporting of adverse events. These results align with previous FAERS-based studies. Moreover, other spontaneous reporting analyses have identified significant safety signals for ibuprofen, including renal injury and gastrointestinal bleeding [26,27]. These findings underscore the need for continued caution when recommending ibuprofen for non-prescription use, especially in populations at higher risk.

This descending trend noted at the beginning of the COVID-19 pandemic could be explained as an effect of studies that initially indicated ibuprofen and the other NSAIDs increased the expression of ACE2, facilitating COVID-19 infection and affecting disease outcomes, although further research proved ibuprofen was not associated with worse clinical outcomes in patients with COVID-19 [28,29,30,31,32]. During the COVID-19 pandemic, NSAIDs have been evaluated for potential therapeutic repositioning, highlighting the importance of continuous pharmacovigilance [32,33,34]; in this context, Xianfang et al. demonstrated that computational and pharmacovigilance-driven approaches can effectively identify potential drug-repurposing candidates, further emphasizing the need for ongoing safety monitoring of widely used drugs such as NSAIDs [34]. Regarding sex distribution, the highest percentage of spontaneous reports was observed in females (47.0%), and the reported adverse event rate in males was 29.9%. Apparently, the analgesic effect was predominant in men, although no pharmacokinetic discrepancies were observed [35]. The presumed sex-related difference in nociception could be attributed to the estrogenic effects on the nervous system, resulting in an increased pain impulse transmission [36]. A higher tendency of reporting adverse events in women than in men could also be arguable.

For the age category, the highest percentage of reports was recorded for the 18–64 years age group (31.1%). The lack of documented patient age was noticed in a relatively large number of cases (45%). In the over 65 years age group, 13% of the total reported cases were recorded. It is important to carefully monitor the risk-benefit ratio in older populations, given the risk of polypathologies and potential drug interactions [37]. Moreover, a study conducted using data from the French pharmacovigilance database highlighted the fact that, of the OTC medicines, ibuprofen was the most frequently linked to severe ADRs in the pediatric population, this aspect raising serious signs of concern [38].

The current study also highlighted the discrepancy between the number of reports and the administration route. As expected, the oral route accounted for the most reported cases (35.36%). Surprisingly, in numerous cases, the administration route was omitted (63.59%). Although transplacental and transmammary routes account for a low percentage (0.52% and 0.02%, respectively), ADRs have still been reported. Thus, ADRs may occur during fetal intrauterine development or in the infant after birth. Therefore, the current work calls attention to the development of ADRs via the transmammary route, although the excreted quantity of ibuprofen in breast milk is low (less than 1 mg/day), out of a 1600 mg/day dose [39,40]. The risk of ADR development as a result of ibuprofen administration over the duration of the pregnancy arises especially from self-medication in yet to be confirmed pregnancies or inadequate treatment. Due to their mechanism of action, NSAID administration is linked with a multitude of possible ADRs in the fetus (neurological, renal, pulmonary, gastrointestinal and cardiovascular damage, i.e., after 30 weeks, oligohydramnios and premature ductus arteriosus closure) [41,42].

According to the present findings, the adverse events of ibuprofen reporting an unfavorable outcome were 30%, and 4.3% of the reported cases were related to death. In a study of the FAERS database conducted by Eugene, analyzing reports from the 2014–2017 period in the adolescent age group (12–17 years), it was revealed that ibuprofen was correlated with the highest rate of unfavorable outcome—suicide [43]. In a retrospective analysis of the United States National Poison Data System, regarding the exposure to intentional overdose (suicide attempt) of acetaminophen and ibuprofen by adolescents in the US during the period 2017–2022, Weigel et al. stated that ibuprofen was connected to central nervous system depression and metabolic acidosis [44]. A congenital anomaly was encountered in 0.3% of cases; the rate was not significantly increased, though the severe implications and the availability of ibuprofen as an OTC should be taken into consideration.

Similar to other scientific evidence [45,46,47], the analyzed data from FAERS emphasized that initial or prolonged hospitalization of the patient was necessary in 24.3% of cases. This elevated percentage could impose another burden on the healthcare system.

In the present study, the top 30 signals with the most records in FAERS were product use in an unapproved indication, hypersensitivity, drug effectiveness for an unapproved indication, and vomiting. Applying the disproportionality analysis, the study pointed out that 17 of the top 30 signals were possible ADRs associated with the active substance ibuprofen. The most notable were gastrointestinal disorders (upper abdominal pain, abdominal discomfort, and gastrointestinal hemorrhage), immune system disorders (hypersensitivity, urticaria, angioedema, and anaphylactic reaction), renal disorders (acute kidney injury), and psychiatric disorders (suicide attempt and somnolence). Hypersensitivity reactions associated with NSAIDs may involve genetic predisposition and immune-mediated mechanisms [48,49]. Tang et al. provided evidence that genetic polymorphisms in immune-regulatory genes contribute to individual susceptibility to allergic and hypersensitivity reactions, supporting the immunologic mechanisms discussed for NSAID-associated adverse events [49]. Additionally, the current findings investigated the distribution of signals according to their likelihood of being connected to ADRs, noting that 20% of the total recorded signals for ibuprofen in FAERS were positive signals, while 80% were false signals.

The signals with the highest chi-square value were regarded as the strongest positive signals. Factoring this value, ADRs were identified for the top 30 strongest positive signals, belonging to the categories of gastrointestinal, allergic, renal, respiratory, metabolic (metabolic acidosis), neurological (aseptic meningitis), psychiatric (suicide attempt), as well as product-related issues (failure of child-resistant mechanism for pharmaceutical product and poor-quality drug administered) or procedural complications (product administered to patient of inappropriate age, accidental exposure to product by child, intentional overdose, product use in unapproved indication or drug effective for unapproved indication).

PGE2 increases the synthesis of mucus that protects the stomach wall against hydrochloric acid [50,51]. NSAIDs interact with phospholipids, thereby initiating biochemical changes that modify the gastrointestinal barrier. By inhibiting the PGE2 synthesis, NSAIDSs could induce mucosal damage, leading to erosions, ulcerations, hemorrhages, structural losses or even perforation [52].

In the sphere of hypersensitivity (allergic and cutaneous reactions), ibuprofen was linked to the most ADRs, including urticaria, angioedema, anaphylaxis, vasculitis, Stevens–Johnson syndrome, and photosensitivity. Reactions to a single NSAID are assumed to be mediated by specific IgE antibodies directed towards the antigenic determinants present within the medication. Patients with this type of immune response could also react to products with a similar chemical structure to that of the inducer drug. Thus, ibuprofen cross-reactions can be found for other compounds from chemically related groups such as naproxen, flurbiprofen, fenoprofen, ketoprofen, etc. Hypersensitivity reactions seem to have COX-1 inhibition as a mechanism, leading to arachidonic acid metabolism deviation towards the 5-lipooxygenase pathway and to the excessive production of cysteinyl leukotrienes, according to the theory proposed by Szczeklik [53]. Furthermore, single-dose ibuprofen-induced Stevens–Johnson syndrome has been scientifically reported, with symptoms manifesting after the administration of a higher dose—800 mg ibuprofen [54]. Moreover, ibuprofen-induced hypersensitivity syndrome could lead to an unpredictable, idiosyncratic type B reaction and could present a significant preoccupation in clinical practice, having been also mentioned in the scientific literature [55].

With regards to the neurological reactions, cases of aseptic meningitis are likewise present within the literature, for instance, Kalfoutzou et al. discussed a case of aseptic meningitis associated with ibuprofen in a 54-year old patient after the administration of a 400 mg ibuprofen dose [56]. Pereira et al. also noted a case report from the pediatric population group, a patient aged 15 [57]. Migraine was accompanied by excessive use of NSAIDs for several pain-related conditions, the prolonged administration causing a rebound effect, with the appearance of migraines [58,59]. On the other hand, regarding the suicidal behaviors correlated with ibuprofen, controversial data have been recorded throughout the scientific literature. Therefore, in some scientific cases, the potential reduction of inflammation at the central level (through the mechanism of action of NSAIDs) could lead to the conclusion that NSAIDs decrease suicidal ideation and depression [60]. Other scientific data also support this hypothesis [48], emphasizing the fact that the physio-pathological modifications (inflammation, oxidative and nitrosative stress, and mitochondrial dysfunctions) on account of major neuropsychiatric disorders (depression, schizophrenia, Alzheimer’s disease, etc.) could be countered by aid of an NSAID class representative, such as aspirin [61]. Nevertheless, an exploratory pharmacovigilance study of FAERS database entries, evaluating the 20 most common medications associated with suicidal ideation and self-harm, revealed that ibuprofen displayed a significant reporting frequency of neuropsychiatric adverse events [62].

The results of our study referring to the renal impairment produced by ibuprofen are in concordance with the literature that highlighted the role of PGs (e.g., PGE2 and PGI2) in maintaining renal blood flow. By inhibiting COX-1 and COX-2, PG biosynthesis is affected, leading to membrane damage, reduced pore size, and decreased podocyte density. As a consequence, renal impairment may occur [63].

Study strengths consisted in the large extension of the study, both in terms of time and geography, thus ensuring the diversity and large number of patients, clearly superior to those that could be achieved by means of controlled clinical trials and with a limited number of patients.

### Study Limitations

The centralized post-authorization safety monitoring of medication is essential, although the current reporting systems exhibit several well-recognized limitations [64]. On one hand, there is the lack of denominator and, on the other hand, the number of submitted reports compared to the occurrence may be reduced by the underreporting phenomenon, especially for mild adverse reactions, or increased during certain time periods, as by the Weber effect and notoriety bias. The heterogeneous nature of the safety reports, in which some data fields are inconsistently completed or left missing, may distort signal detection and hinder the accurate estimation of adverse drug reaction incidence using the FAERS database. Furthermore, confounding by indication cannot be fully excluded and reported reactions may be related to the underlying disease rather than to the medication itself. A major impediment stems from the impossibility of establishing a cause–effect relationship between the exposure to a medication and the reported event [65,66]. Despite these limitations, to assess the robustness of our findings, sensitivity analyses were conducted using multiple complementary disproportionality measures, including the reporting odds ratio (ROR) and the proportional reporting ratio (PRR). The consistency of signals detected across these methods supports the robustness of the observed associations. As disproportionality analyses involve multiple testing across numerous drug–event pairs, results derived from the FAERS database should be interpreted with caution and considered targeted safety signals that merit subsequent clinical evaluation rather than confirmatory evidence of causality.

## 4. Materials and Methods

The Food and Drug Administration (FDA) of the United States of America (US) possesses a reporting system of potential ADRs, referred to as the FDA Adverse Events Reporting System (FAERS), that includes national and international reports for all drugs marketed in the US.

The OpenVigil 2.1 tool [67] was used to retrieve and analyze adverse event (AE) reports for ibuprofen as the active pharmaceutical ingredient from the FAERS database due to the availability of cleaned and validated data [6].

Ibuprofen was queried in OpenVigil 2.1 using the active substance name, with the drug role restricted to “primary suspect”. The analysis included all FAERS reports recorded between 2004 and 2024. Deduplication was performed by retaining only the most recent version of each case, based on both “case_ID” and “primary_id”, thereby avoiding duplicate counts of follow-up reports. Adverse events were coded according to the Medical Dictionary for Regulatory Activities (MedDRA), version 28.1, and analyses were conducted at the Preferred Term (PT) level.

**Figure 7:**
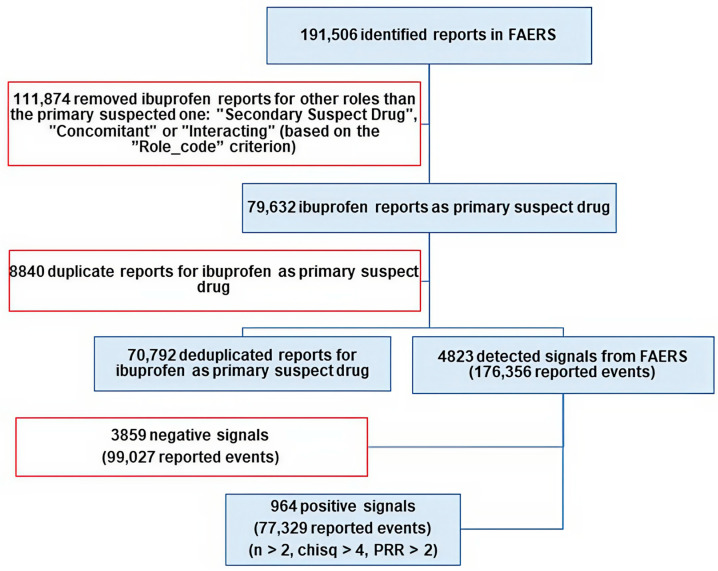
Flowchart of the analysis of adverse events reported for ibuprofen in the FAERS database up to 31 December 2024. PRR—proportional reporting ratio. Excluded data are framed in red [6,67].

Initially, a total of 79,632 reports listing ibuprofen as the primary suspect drug were identified. To address potential duplicate entries, reports were subsequently deduplicated based on the unique “case_ID” criterion, in accordance with established FAERS methodology [68]. Following the deduplication process, 70,792 unique cases remained and were included in the final analysis. All signal detection analyses, figures, and tables were generated using this final deduplicated dataset (Figure 7).

Initially, the descriptive analysis followed the chronological evolution of the submission of these individual case safety reports (ICSRs) to FAERS until 2024 and, eventually, their origin. Demographic characteristics of patients (sex and age) and ibuprofen administration route, as well as reports with unfavorable outcomes, were further analyzed. The category of reports with unfavorable outcomes included: death, initial or prolonged hospitalization of the patient, life-threatening events, disability, and congenital anomaly that required intervention to prevent permanent impairment/damage of the patient’s condition. Reports indicating “lack of efficacy” (PT: drug ineffective) were identified and analyzed separately from the other ADRs to avoid conflating safety with effectiveness. Only reports meeting the criteria for ADRs were included in the signal detection analysis.

Signal assessment focused on the top 30 entries with the highest number of reports. Disproportionality screening was performed using Evans’ criteria, including a minimum number of reports (n ≥ 2), a chi-square value greater than 4 (χ^2^ ≥ 4), and a proportional reporting ratio (PRR) greater than 2 (PRR > 2) [69]. The reporting odds ratio (ROR) was subsequently used to quantify signal strength and assess the magnitude of disproportionality. For all ROR estimates, 95% confidence intervals (95% CI) were calculated, and signal robustness was evaluated using the lower bound of the confidence interval.

The top 30 positive signals with the highest chi-square values were considered the strongest. Adverse events were classified as “Potential ADRs” if they met all Evans’ criteria and were consistent with the known pharmacology of ibuprofen and clinical guidelines. Events not meeting all criteria were categorized as “No” for potential ADR, even if some are recognized ibuprofen-associated ADRs, to ensure transparency and reproducibility of the classification. The disproportionality analysis approach is consistent with recent FAERS-based studies. Liu et al. (2025) demonstrated the applicability and robustness of disproportionality analysis for detecting safety signals from FAERS database associated with immunomodulatory therapies, supporting the methodological approach used in the present study [70].

Data uploaded until 31 December 2024 were extracted and processed using the Microsoft Excel for Microsoft 365 MSO (Version 2503). Ethical approval was not required for this study because all analyses were conducted using the publicly available FAERS database, which contains fully anonymized and de-identified adverse event reports. Similar other pharmacovigilance studies using FAERS or EudraVigilance data reports were performed [71,72].

## 5. Conclusions

Ibuprofen continues to be one of the popularly used NSAIDs, confirmed by the large number of reports recorded in FAERS, across all age groups. Reported adverse events fall into the characteristic classes for ibuprofen, positive signals having been highlighted for events pertaining to the gastrointestinal sphere, allergic reactions, procedural complications, and renal disorders. The disproportionality analysis led to the identification of possible ADRs, such as drug hypersensitivity, acute kidney injury, urticaria, upper abdominal pain, and angioedema. The results of the study should be interpreted while accommodating the inherent limitations of the collected data as a consequence of spontaneous reporting. The results of the present study underline the possible risks related to ibuprofen use and underscore the importance of a responsible and clinically well-founded administration.

## Figures and Tables

**Table 1:** Administration routes stated in the reports recorded in FAERS for ibuprofen (n = 70,792).

Administration Route	Number of Reports (n)	Percentage (%)
Oral	25,035	35.36%
Transplacental	367	0.52%
Intravenous	153	0.22%
Unspecified	29,866	42.19%
Unknown	15,126	21.37%
Other	245	0.34%

Unknown: route of administration was not reported; Unspecified: the report does not provide further details; Other: rare (<0.10%) or unclassified administration routes, including buccal, oropharyngeal, sublingual, dental, respiratory (inhalation), topical, cutaneous, transdermal, rectal, parenteral, intramuscular, intratracheal, intratumor, intrauterine, endocervical, auricular, nasal, ophthalmic, intraocular, and transmammary.

**Table 2:** Adverse events with over 1000 reports.

Reported Adverse Event	Potential ADR	Number of Reports	Percentage of Total Reports
Drug ineffective	No	8772	5.00%
Product use in unapproved indication	Yes	3569	2.03%
Drug hypersensitivity	Yes	3407	1.94%
Drug effective for unapproved indication	Yes	2459	1.40%
Vomiting	No	2447	1.40%
Nausea	No	2166	1.23%
Overdose	Yes	1912	1.09%
Urticaria	Yes	1828	1.04%
Acute kidney injury	Yes	1782	1.02%
Off-label use	No	1772	1.01%
Dyspnea	No	1724	0.98%
Intentional overdose	Yes	1664	0.95%
Abdominal pain upper	Yes	1638	0.93%
Angioedema	Yes	1636	0.93%
Toxicity to various agents	Yes	1466	0.84%
Somnolence	Yes	1463	0.83%
Rash	No	1394	0.79%
Dizziness	No	1353	0.77%
Abdominal discomfort	Yes	1349	0.77%
Hypersensitivity	Yes	1346	0.77%
Pruritus	No	1292	0.74%
Headache	No	1289	0.73%
Incorrect dose administered	No	1229	0.70%
Condition aggravated	No	1199	0.68%
Diarrhea	No	1092	0.62%
Suicide attempt	Yes	1070	0.61%
Gastrointestinal hemorrhage	Yes	1067	0.61%
Intentional product misuse	Yes	1061	0.60%
Anaphylactic reaction	Yes	1052	0.60%
Malaise	No	1048	0.60%

**Table 3:** Reporting odds ratio representation for the strongest positive signals.

Positive Signal	Chi_Squared	ROR	95% CI Lower Bound	95% CI Upper Bound
failure of child-resistant product closure	4094.09	182.16	121.30	273.54
NSAID-exacerbated respiratory disease	8783.94	161.43	124.14	209.93
aspirin-exacerbated respiratory disease	6552.15	68.50	56.27	83.38
bronchopulmonary dysplasia	5193.93	56.16	46.01	68.55
glomerulonephritis minimal lesion	3121.73	43.44	34.70	54.38
renal tubular acidosis	5647.44	34.57	29.80	40.10
drug effective for unapproved indication	51,425.10	26.63	25.52	27.80
lip oedema	6096.84	26.11	23.09	29.53
poor-quality drug administered	6582.94	20.82	18.75	23.12
meningitis aseptic	3248.81	18.97	16.46	21.85
duodenal ulcer	4529.49	17.55	15.65	19.69
product administered to patient of inappropriate age	4203.42	16.74	14.90	18.80
accidental exposure to product by child	4546.16	14.85	13.38	16.48
gastric ulcer	6357.27	12.59	11.62	13.64
upper gastrointestinal hemorrhage	4854.22	11.70	10.72	12.78
angioedema	14,491.17	11.54	10.97	12.13
toxic epidermal necrolysis	3639.32	10.40	9.47	11.44
Melaena	5029.26	10.37	9.57	11.23
oral discomfort	3185.19	10.27	9.29	11.35
gastric hemorrhage	3066.28	10.20	9.21	11.29
hematemesis	5193.09	9.95	9.21	10.75
tubulointerstitial nephritis	4011.49	9.43	8.66	10.26
metabolic acidosis	4008.25	7.55	7.01	8.13
intentional overdose	8798.20	7.50	7.14	7.88
anaphylactic reaction	4785.54	6.64	6.24	7.06
suicide attempt	3231.44	4.95	4.66	5.26
drug hypersensitivity	9675.03	4.85	4.69	5.03
product use in unapproved indication	10,036.44	4.83	4.67	5.00
acute kidney injury	3713.88	3.93	3.75	4.12
urticaria	3722.68	3.88	3.70	4.06

## Data Availability

The original contributions presented in this study are included in the article. Further inquiries can be directed to the corresponding author.

## References

[1] The Top 300 of 2022. https://clincalc.com/DrugStats/Top300Drugs.aspx.

[2] Bookstaver B., Miller A.D., Norris L.B., Rudisill C.N. (2010). Intravenous Ibuprofen: The First Injectable Product for the Treatment of Pain and Fever. J. Pain Res..

[3] Prasaja B., Harahap Y., Sandra M., Iskandar I., Lusthom W., Cahyaningsih P. (2022). Rectal Administration of Ibuprofen: Comparison of Enema and Suppository Form. Drug Res..

[4] Moore N. (2007). Ibuprofen: A Journey from Prescription to over-the-Counter Use. J. R. Soc. Med..

[5] Irvine J., Afrose A., Islam N. (2018). Formulation and Delivery Strategies of Ibuprofen: Challenges and Opportunities. Drug Dev. Ind. Pharm..

[6] FDA Adverse Event Reporting System (FAERS) Public Dashboard for Drugs and Biologics—FDA Adverse Event Reporting System (FAERS) Public Dashboard for Drugs and Biologics | Sheet—Qlik Sense. https://fis.fda.gov/sense/app/95239e26-e0be-42d9-a960-9a5f7f1c25ee/sheet/33a0f68e-845c-48e2-bc81-8141c6aaf772/state/analysis.

[7] Gómez-Acebo I., Dierssen-Sotos T., De Pedro M., Pérez-Gómez B., Castaño-Vinyals G., Fernández-Villa T., Palazuelos-Calderón C., Amiano P., Etxeberria J., Benavente Y. (2018). Epidemiology of Non-Steroidal Anti-Inflammatory Drugs Consumption in Spain. The MCC-Spain Study. BMC Public Heal..

[8] Young C., Eggleston W. (2024). Ibuprofen. Encycl. Toxicol. Fourth Ed..

[9] Sobhani K., Li J., Cortes M. (2023). Nonsteroidal Anti-Inflammatory Drugs (NSAIDs). First Aid Perioperative Ultrasound.

[10] Stiller C.O., Hjemdahl P. (2022). Lessons from 20 Years with COX-2 Inhibitors: Importance of Dose-Response Considerations and Fair Play in Comparative Trials. J. Intern. Med..

[11] Sostres C., Gargallo C.J., Arroyo M.T., Lanas A. (2010). Adverse Effects of Non-Steroidal Anti-Inflammatory Drugs (NSAIDs, Aspirin and Coxibs) on Upper Gastrointestinal Tract. Best Pract. Res. Clin. Gastroenterol..

[12] Kowalski M.L., Asero R., Bavbek S., Blanca M., Blanca-Lopez N., Bochenek G., Brockow K., Campo P., Celik G., Cernadas J. (2013). Classification and Practical Approach to the Diagnosis and Management of Hypersensitivity to Nonsteroidal Anti-Inflammatory Drugs. Allergy.

[13] Bally M., Dendukuri N., Rich B., Nadeau L., Helin-Salmivaara A., Garbe E., Brophy J.M. (2017). Risk of Acute Myocardial Infarction with NSAIDs in Real World Use: Bayesian Meta-Analysis of Individual Patient Data. BMJ.

[14] Yancy C.W., Jessup M., Bozkurt B., Butler J., Casey D.E., Drazner M.H., Fonarow G.C., Geraci S.A., Horwich T., Januzzi J.L. (2013). 2013 ACCF/AHA Guideline for the Management of Heart Failure: A Report of the American College of Cardiology Foundation/American Heart Association Task Force on Practice Guidelines. Circulation.

[15] Shao Q.H., Yin X.D., Liu H.X., Zhao B., Huang J.Q., Li Z.L. (2021). Kidney Injury Following Ibuprofen and Acetaminophen: A Real-World Analysis of Post-Marketing Surveillance Data. Front. Pharmacol..

[16] Auriel E., Regev K., Korczyn A.D. (2014). Nonsteroidal Anti-Inflammatory Drugs Exposure and the Central Nervous System. Handb. Clin. Neurol..

[17] Buciuman C.A., Dobrea C.M., Butuca A., Frum A., Gligor F.G., Botea M.O., Vicaș L.G., Mureșan M.E., Gligor O., Maghiar F. (2025). Pharmacovigilance Insights into Ibuprofen’s Neuropsychiatric Safety: A Retrospective Analysis of EudraVigilance Reports. Pharmaceuticals.

[18] Romano A., Valluzzi R.L., Alvarez-Cuesta E., Ansotegui I., Asero R., Barbaud A., Bartra J., Bavbek S., Cahill K.N., Demoly P. (2025). Updating the Classification and Routine Diagnosis of NSAID Hypersensitivity Reactions: A WAO Statement. World Allergy Organ. J..

[19] Li D., Wang D., Huang G., Liu Q., Wang Y., Zhang R., Liu S., Du Q. (2026). Drug-Induced Liver Injury: A Real-World Pharmacovigilance Study Using the FDA Adverse Event Reporting System Database (2004–2024). Ann. Hepatol..

[20] Sriuttha P., Sirichanchuen B., Permsuwan U. (2018). Hepatotoxicity of Nonsteroidal Anti-Inflammatory Drugs: A Systematic Review of Randomized Controlled Trials. Int. J. Hepatol..

[21] Buciuman C.A., Dobrea C.M., Butuca A., Frum A., Gligor F.G., Gligor O., Vicaș L.G., Morgovan C. (2025). Exploring the Cardiovascular Safety Profile of Ibuprofen: Insights from EudraVigilance Database. Pharmaceuticals.

[22] Help to Shape the MedDRA Terminology | MedDRA. https://www.meddra.org/.

[23] Jiao X.F., Pu L., Lan S., Li H., Zeng L., Wang H., Zhang L. (2024). Adverse Drug Reaction Signal Detection Methods in Spontaneous Reporting System: A Systematic Review. Pharmacoepidemiol. Drug Saf..

[24] Vassallo F., Martinelli M., Varcamonti L., Buono P. (2025). Adverse Reactions to Acetaminophen and Ibuprofen in Pediatric Patients: A Narrative Review. Ital. J. Pediatr..

[25] Paul A.E., Sasidharanpillai S. (2025). Role of Pharmacovigilance in Drug Safety Monitoring. Indian Dermatol. Online J..

[26] Xu H., Cao J., Zhang H., Fei F., Tang D., Liu D., Luo D. (2025). Renal Injury in NSAIDs: A Real-World Analysis Based on the FAERS Database. Int. Urol. Nephrol..

[27] Amatya E., Fois R., Williams K.A., Pont L.G. (2020). Potential for Detection of Safety Signals for Over-the-Counter Medicines Using National ADR Spontaneous Reporting Data: The Example of OTC NSAID-Associated Gastrointestinal Bleeding. Pharmacy.

[28] Rochette L., Ghibu S. (2021). Mechanics Insights of Alpha-Lipoic Acid against Cardiovascular Diseases during COVID-19 Infection. Int. J. Mol. Sci..

[29] Abu Esba L.C., Alqahtani R.A., Thomas A., Shamas N., Alswaidan L., Mardawi G. (2021). Ibuprofen and NSAID Use in COVID-19 Infected Patients Is Not Associated with Worse Outcomes: A Prospective Cohort Study. Infect. Dis. Ther..

[30] Fang L., Karakiulakis G., Roth M. (2020). Are Patients with Hypertension and Diabetes Mellitus at Increased Risk for COVID-19 Infection?. Lancet Respir. Med..

[31] Kushner P., McCarberg B.H., Grange L., Kolosov A., Haveric A.L., Zucal V., Petruschke R., Bissonnette S. (2022). The Use of Non-Steroidal Anti-Inflammatory Drugs (NSAIDs) in COVID-19. NPJ Prim. Care Respir. Med..

[32] Laughey W., Lodhi I., Pennick G., Smart L., Sanni O., Sandhu S., Charlesworth B. (2023). Ibuprofen, Other NSAIDs and COVID-19: A Narrative Review. Inflammopharmacology.

[33] Micallef J., Soeiro T., Jonville-Béra A.P. (2020). COVID-19 and NSAIDs: Primum Non Nocere. Therapies.

[34] Tang X., Cai L., Meng Y., Xu J.L., Lu C., Yang J. (2021). Indicator Regularized Non-Negative Matrix Factorization Method-Based Drug Repurposing for COVID-19. Front. Immunol..

[35] Farkouh A., Riedl T., Gottardi R., Czejka M., Kautzky-Willer A. (2020). Sex-Related Differences in Pharmacokinetics and Pharmacodynamics of Frequently Prescribed Drugs: A Review of the Literature. Adv. Ther..

[36] Farkouh A., Baumgärtel C., Gottardi R., Hemetsberger M., Czejka M., Kautzky-Willer A. (2021). Sex-Related Differences in Drugs with Anti-Inflammatory Properties. J. Clin. Med..

[37] Wongrakpanich S., Wongrakpanich A., Melhado K., Rangaswami J. (2018). A Comprehensive Review of Non-Steroidal Anti-Inflammatory Drug Use in The Elderly. Aging Dis..

[38] Durrieu G., Maupiler M., Rousseau V., Chebane L., Montastruc F., Bondon-Guitton E., Montastruc J.L. (2018). Frequency and Nature of Adverse Drug Reactions Due to Non-Prescription Drugs in Children: A Retrospective Analysis from the French Pharmacovigilance Database. Paediatr. Drugs.

[39] Pregnancy, Breastfeeding and Fertility While Taking Ibuprofen and Codeine—NHS. https://www.nhs.uk/medicines/ibuprofen-and-codeine-nurofen-plus/pregnancy-breastfeeding-and-fertility-while-taking-ibuprofen-and-codeine/.

[40] Ibuprofen. https://www.ncbi.nlm.nih.gov/books/NBK582759/.

[41] Antonucci R., Zaffanello M., Puxeddu E., Porcella A., Cuzzolin L., Dolores Pilloni M., Fanos V. (2012). Use of Non-Steroidal Anti-Inflammatory Drugs in Pregnancy: Impact on the Fetus and Newborn. Curr. Drug Metab..

[42] Chen X., Yang Y., Chen L., Wang K. (2024). Pregnancy Outcomes and Birth Defects in Offspring Following Non-Steroidal Anti-Inflammatory Drugs Exposure during Pregnancy: A Systematic Review and Meta-Analysis. Reprod. Toxicol..

[43] Eugene A.R., Eugene B. (2018). An Opportunity for Clinical Pharmacology Trained Physicians to Improve Patient Drug Safety: A Retrospective Analysis of Adverse Drug Reactions in Teenagers. F1000Research.

[44] Weigel B., Adams A., Wahrenbrock T., Wahl M. (2024). Adolescent Acetaminophen and Ibuprofen Self-Poisoning, 2017–2022. Pediatr. Emerg. Care.

[45] Hunter Erin NSAIDs Linked to Thousands of Heart Failure Hospitalizations Among Patients with Type 2 Diabetes | Pharmacy Times. https://www.pharmacytimes.com/view/nsaids-linked-to-thousands-of-heart-failure-hospitalizations-among-patients-with-type-2-diabetes.

[46] Su L., Li Y., Xu R., Luo F., Gao Q., Chen R., Cao Y., Nie S., Xu X. (2021). Association of Ibuprofen Prescription With Acute Kidney Injury Among Hospitalized Children in China. JAMA Netw. Open.

[47] Moon K.T. (2009). NSAID Use Linked to Increased Cardiovascular Risk. Am. Fam. Physician.

[48] Sánchez-Borges M., Caballero-Fonseca F., Capriles-Hulett A., González-Aveledo L. (2010). Hypersensitivity Reactions to Nonsteroidal Anti-Inflammatory Drugs: An Update. Pharmaceuticals.

[49] Tang L., Chen Y., Xiang Q., Xiang J., Tang Y., Li J. (2020). The Association between IL18, FOXP3 and IL13 Genes Polymorphisms and Risk of Allergic Rhinitis: A Meta-Analysis. Inflamm. Res..

[50] Sohail R., Mathew M., Patel K.K., Reddy S.A., Haider Z., Naria M., Habib A., Abdin Z.U., Razzaq Chaudhry W., Akbar A. (2023). Effects of Non-Steroidal Anti-Inflammatory Drugs (NSAIDs) and Gastroprotective NSAIDs on the Gastrointestinal Tract: A Narrative Review. Cureus.

[51] Peng S., Duggan A. (2005). Gastrointestinal Adverse Effects of Non-Steroidal Anti-Inflammatory Drugs. Expert Opin. Drug Saf..

[52] Bjarnason I., Scarpignato C., Holmgren E., Olszewski M., Rainsford K.D., Lanas A. (2018). Mechanisms of Damage to the Gastrointestinal Tract from Nonsteroidal Anti-Inflammatory Drugs. Gastroenterology.

[53] Szczeklik A., Szcuklik A., Respir E. (1990). The Cyclooxygenase Theory of Aspirin-Induced Asthma. Eur. Respir. J..

[54] Alhatemi A.Q.M., Hashim H.T., Al-Tarbosh M.A.S., Abdulhussain R., Hashim A.T. (2024). Single-Dose Ibuprofen Induced Stevens-Johnson Syndrome. Clin. Case Rep..

[55] Nanau R.M., Neuman M.G. (2010). Ibuprofen-Induced Hypersensitivity Syndrome. Transl. Res..

[56] Kalfoutzou A., Petroulakis P., Tsiouri E., Fafoutis T., Mylonakis A., Dimitrakoudi M., Mylonaki M., Piperis C., Mostratou E. (2024). Ibuprofen: The Hidden Culprit Behind Aseptic Meningitis. Cureus.

[57] Pereira Pires S.A., Lemos A.P., Nunes Pereira E.P.M., da Silva Vilar Maia P.A., de Sousa J.P. (2019). Ibuprofen-Induced Aseptic Meningitis: A Case Report. Rev. Paul. Pediatr..

[58] Stopping the Vicious Cycle of Rebound Headaches—Harvard Health. https://www.health.harvard.edu/blog/stopping-the-vicious-cycle-of-rebound-headaches-2019110718180.

[59] Fischer M.A., Jan A. (2023). Medication-Overuse Headache.

[60] Lehrer S., Rheinstein P.H. (2019). Nonsteroidal Anti-Inflammatory Drugs (NSAIDs) Reduce Suicidal Ideation and Depression. Discov. Med..

[61] Berk M., Dean O., Drexhage H., McNeil J.J., Moylan S., O’Neil A., Davey C.G., Sanna L., Maes M. (2013). Aspirin: A Review of Its Neurobiological Properties and Therapeutic Potential for Mental Illness. BMC Med..

[62] Xie W.L., Xiang D.C., Li Y.Y., Ge M.L., Deng A.P. (2025). An Exploratory Study Evaluating the 20 Medications Most Commonly Associated with Suicidal Ideation and Self-Injurious Behavior in the FAERS Database. BMC Pharmacol. Toxicol..

[63] Drożdżal S., Lechowicz K., Szostak B., Rosik J., Kotfis K., Machoy-Mokrzyńska A., Białecka M., Ciechanowski K., Gawrońska-Szklarz B. (2021). Kidney Damage from Nonsteroidal Anti-Inflammatory Drugs-Myth or Truth? Review of Selected Literature. Pharmacol. Res. Perspect..

[64] Imbrici P., De Bellis M., Liantonio A., De Luca A. (2025). Investigating the Benefit-Risk Profile of Drugs: From Spontaneous Reporting Systems to Real-World Data for Pharmacovigilance. Methods Mol. Biol..

[65] Reeves B.N., Masarova L., Abu-Zeinah G., Hunter A.M., Shatzel J.J., Qin A., Yoon C.H., Cai L.Y., Wei Y.F., Mesa R.A. (2025). A Pharmacovigilance Study of Adverse Events Associated with Polycythemia Vera Treatments Using the FDA Adverse Event Reporting System (FAERS) Database. Ann. Hematol..

[66] Chedid V., Vijayvargiya P., Camilleri M. (2018). Advantages and Limitations of the Federal Adverse Events Reporting System in Assessing Adverse Event Reporting for Eluxadoline. Clin. Gastroenterol. Hepatol..

[67] OpenVigil—Open Tools for Data-Mining and Analysis of Pharmacovigilance Data. https://openvigil.sourceforge.net/.

[68] Popa Ilie I.R., Butuca A., Homorodean C., Dobrea C.M., Morgovan C., Frum A., Ghibu S. (2025). Osilodrostat Safety Profile: Findings from Real-World Data in the FAERS Database. J. Clin. Med..

[69] Evans S.J.W., Waller P.C., Davis S. (2001). Use of Proportional Reporting Ratios (PRRs) for Signal Generation from Spontaneous Adverse Drug Reaction Reports. Pharmacoepidemiol. Drug Saf..

[70] Liu B., Zhang W., Chen Y., Han A., Chen D. (2025). Pharmacovigilance Analysis of FcRn Antagonists in the Treatment of Myasthenia Gravis: A Disproportionality Analysis Based on the FAERS Database. Hum. Vaccines Immunother..

[71] Zhu B.K., Chen S.Y., Li X., Huang S.Y., Luo Z.Y., Zhang W. (2025). Real-World Pharmacovigilance Study of Drug-Induced Autoimmune Hepatitis from the FAERS Database. Sci. Rep..

[72] Morgovan C., Dobrea C.M., Butuca A., Arseniu A.M., Frum A., Rus L.L., Chis A.A., Juncan A.M., Gligor F.G., Georgescu C. (2024). Safety Profile of the Trastuzumab-Based ADCs: Analysis of Real-World Data Registered in EudraVigilance. Biomedicines.

